# Nitric oxide promotes epidermal stem cell migration via cGMP-Rho GTPase signalling

**DOI:** 10.1038/srep30687

**Published:** 2016-07-29

**Authors:** Rixing Zhan, Weifeng He, Fan Wang, Zhihui Yao, Jianglin Tan, Rui Xu, Junyi Zhou, Yuzhen Wang, Haisheng Li, Jun Wu, Gaoxing LUO

**Affiliations:** 1 Institute of Burn Research; State Key Laboratory of Trauma, Burn and Combined Injury; Key Laboratory of Proteomics of Chongqing, Southwest Hospital, Third Military Medical University, Chongqing, China

## Abstract

The migration and reepithelization of epidermal stem cells (ESCs) are the most critical processes in wound healing. The gaseous messenger nitric oxide (NO) has multiple biological effects, but its actions on ESCs are poorly understood. In this study, an NO donor, S-nitroso-N-acetylpenicillamine (SNAP), was found to facilitate the *in vitro* migration of human ESCs (huESCs) in both live-imaging and scratch models. In addition, pull-down assays demonstrated that SNAP could activate the small GTPases RhoA and Rac1 of the Rho family, but not Cdc42. Moreover, the effects of SNAP on the migration and F-actin polymerization of ESCs could be blocked by inhibitors of cGMP, PKG, RhoA or Rac1, and by a specific siRNA of RhoA or Rac1, but not by a Cdc42 inhibitor or siRNA. Furthermore, the roles of NO in ESC migration via cGMP-Rho GTPase signalling *in vivo* were confirmed by tracing 5-bromo-2-deoxyuridine (BrdU)-labelled cells in a superficial, partial-thickness scald mouse model. Thus, the present study demonstrated that the NO donor SNAP could promote huESC migration *in vitro*. Furthermore, NO was found to induce ESC migration via cGMP-Rho GTPase RhoA and Rac1 signalling, but not Cdc42 signalling, both *in vivo* and *in vitro*.

Wound healing is a complex process mediated by a variety of factors responsible for the regeneration and reorganization of damaged tissue into its normal architecture. Although the mechanisms of skin repair have not been completely elucidated, many cellular components are known to contribute to the maintenance of skin homeostasis and regeneration^1^,^2^. ESCs that reside in the basal layer of the skin epidermis and hair follicles are essential for skin homeostasis and wound healing^3^,^4^. Under normal conditions, the homeostasis of interfollicular epidermis and hair follicles is maintained by their own distinct stem cells. However, upon skin injury, both stem cell populations are capable of regenerating the two structures. Through fate-mapping experiments, ESCs have been shown to be recruited to the epidermis and to migrate in a linear manner towards the centre of the wound^5^,^6^. However, little is known regarding the mechanisms that stimulate ESC migration during wound repair.

In the past two decades, NO has emerged as a critical molecule for both wound healing^7^,^8^ and the underlying cellular homeostasis^9^,^10^. NO regulation during wound healing depends on the modulation of NO by different cell types involved in this complex reconstruction process^1^,^2^. After skin damage, NO levels increase rapidly and peak 1 day after the initial injury, during the inflammatory phase of wound healing^8^. On the other hand, NO is released by various types of immune and skin cells during wound healing, causing pleiotropic effects. Among its many effects, NO plays a critical role in wound healing by promoting cell migration and proliferation. Our previous study show that NO enhanced human keratinocyte cell (HaCaT) migration^11^. The mechanism of NO on wound healing has been studied by several authors, but its exact signalling is not completely elucidated^12^,^13^. The pathways directly downstream of NO signalling consist primarily of interactions between NO and heme-containing proteins, the most important of which is guanylate cyclase^14^,^15^. The cGMP-synthesizing enzyme, soluble guanylyl cyclase (sGC), is differentially expressed during neuronal development and is a major receptor for NO^16^,^17^. NO- cGMP signal transduction has been shown to regulate the migration of lung cancer cells, endothelial cells, and neural progenitor cells^18^,^19^.

Additionally, small GTP-binding proteins of the Rho family, including RhoA, Rac1, and Cdc42, closely regulate actin-based structure formation and subsequentially cell migration^20^,^21^. Rho GTPases are important regulators of cytoskeletal dynamics, and each GTPase contributes to cell motility by regulating actin cytoskeletal rearrangements^22^. Zhou *et al*. reported that NO causes macrophage migration via the HIF-1-stimulated small GTPases Cdc42 and Rac1^23^. Fujita *et al*. reported that NO increases the invasion of pancreatic cancer cells via the activation of RhoA pathways after carbon ion irradiation^24^. Therefore, we hypothesized that NO may stimulate ESC migration via cGMP-Rho GTPase signal transduction.

Our previous study showed that the NO donor sodium nitroprusside (SNP) could promote HaCat migration *in vitro* through cGMP signalling^11^. The present study elucidated the potential effects of NO on ESC migration and identified the underlying mechanisms *in vitro*. In addition, we investigated the roles of NO in ESC migration both *in vivo*. Our founding might richen the mechanism of NO in wound healing.

## Results

### Culture and characterization of human epidermal stem cells (huESCs) *in vitro*

Stem cells are defined by their unique slow cycling and high proliferative potential^25^. Basal keratinocytes with the cell surface phenotypes α_6_^bri^ and CD71^dim^ exhibit many predicted characteristics of huESCs^26^. In addition, the high level of β_1_ integrin expression in huESCs makes their enrichment simple via rapid adhesion to type IV collagen^26^,^27^. This method was used to isolate ESCs in our experiments. Approximately 50% of these rapidly adherent (RA) cells formed large colonies and grew to confluence within 2 weeks. The expression of β_1_ integrin and CK19 in the RA cells was examined using immunofluorescence staining. Almost all of the cells from a single colony were positive for both β_1_ integrin and CK19. The freshly isolated cells were composed of an α_6_^bri^/CD71^dim^-positive stem cell fraction of 94.3 ± 4.36%. These cells had a strong clonogenic capacity, with a mean colony-forming efficiency (CFE) of 33.1 ± 4.8% at passage 2. The clonogenic capacity and expression of ESC specific markers indicated that the prepared cells were ESCs (data not shown).

### The effect of NO on the migration of isolated huESCs *in vitro*

**Figure 1:**
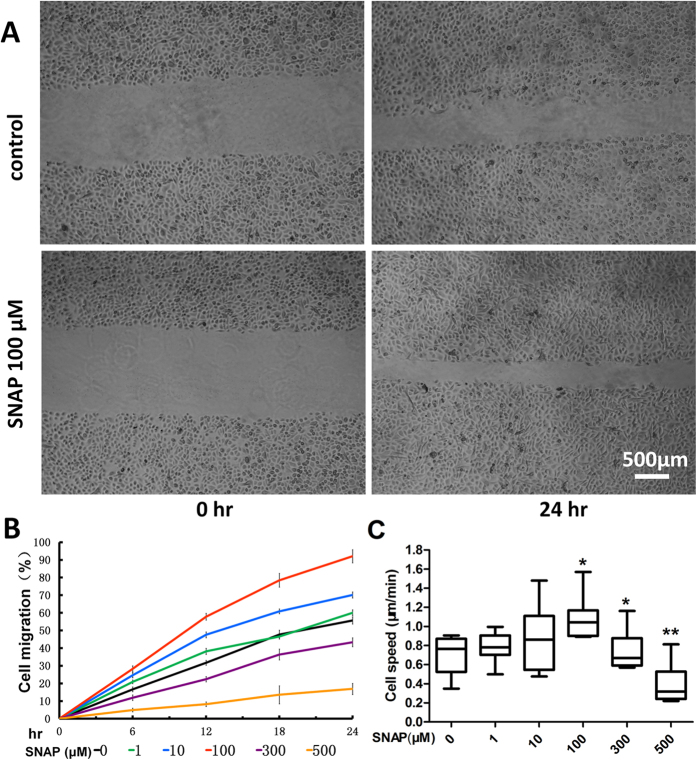
SNAP promoted huESC migration *in vitro*. (**A**) SNAP-stimulated closure of an artificial wound. Wounds were established in confluent monolayers of huESCs, as described in the Materials and methods section. The wounded monolayers were cultured for 24 h after the addition of various concentrations of SNAP. Time-lapse imaging of the wound edges was performed under an inverted phase-contrast microscope. The results shown are representative of three independent experiments. Scale bars, 500 μm. (**B**) Graphical analysis of (**A**). The residual areas were quantified using ImageJ software. The migration rates were calculated using the following equation: (initial area - residual area)/initial area × 100%. The data are expressed as the mean ± SD of values from three independent experiments, each in duplicate (n = 3) (general univariate linear model). Cell migration was quantified as a percentage of the open wound area. (**C**) The average cell speed was determined. The results are shown as a boxplot; the line marks the median speed, and the whiskers mark the minimum and maximum data points. The data are representative of three independent experiments (one-way analysis of variance); *P < 0.05 versus the control, **P < 0.01 versus the control.

In the scratch model, the migration of the isolated huESCs could be enhanced by SNAP in a concentration-dependent manner, as shown in Fig. 1A,B. Compared with the migration of the control group (48.8 ± 2.7%) at hour 12 h after culture, the migration of the cells stimulated with 100 μmol/L SNAP reached a peak of 82.1 ± 15.8% (P < 0.01). However, 500 μmol/L SNAP inhibited cell migration compared with the control. SNAP concentration below 500 μM was not cytotoxic to ESCs; however, when a SNAP concentration of 1000 μM was used, nearly 50% of the ESCs became unviable (Supplementary Fig. 1). The time-lapse cell motility assay was conducted using single cells. In the presence of SNAP, cell motility changed in a dose-dependent manner (Fig. 1C). Exposing ESCs to SNAP (1–100 μM) for 24 h significantly facilitated ESC motility. However, 500 μM SNAP inhibited cell motility (Fig. 1C). These results hint that exogenous NO exerts a biphasic effect on ESC migration. Low doses (1–100 μM) of NO donors (i.e., SNAP) induce migration signals, whereas high doses (300–500 μM, in our study) produce inhibitory effects.

### NO activates the Rho GTPases Rac1 and RhoA, but not Cdc42, in ESCs via cGMP-mediated signal transduction

**Figure 2:**
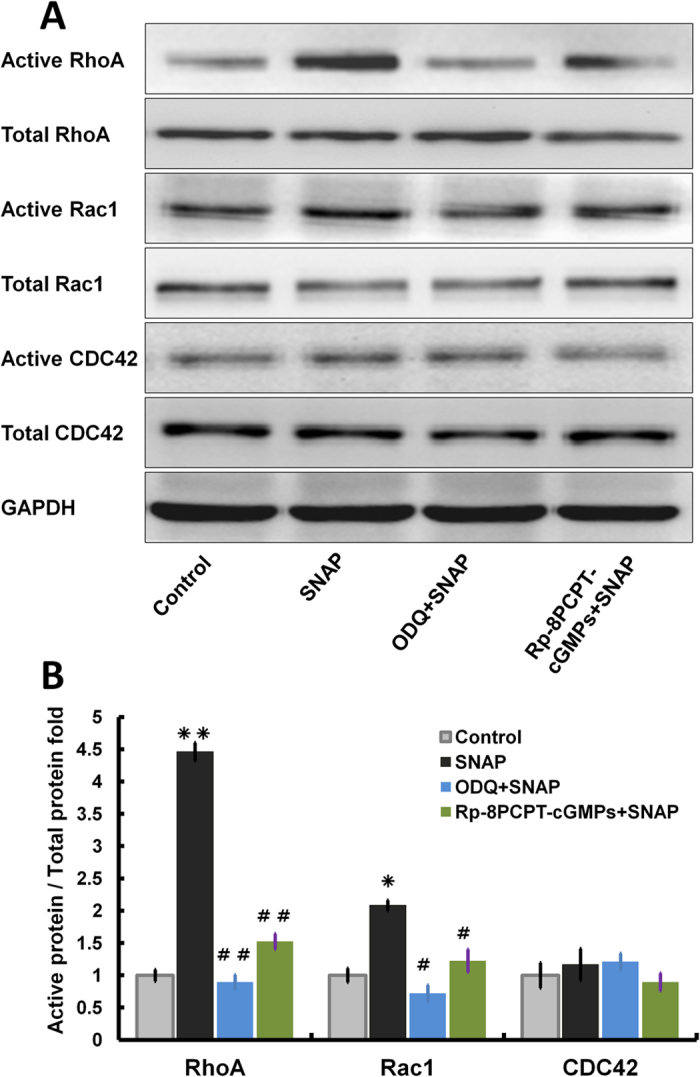
NO activated Rho GTPase via cGMP/PKG. Growth factor-starved ESCs were stimulated with 100 μM SNAP for 24 h with or without pretreatment for 10 min with 1 μM of ODQ as a cGMP inhibitor or 50 μM Rp-8-pcpt-cGMPs as a PKG inhibitor. (**A**) Active Rho-GTPase was detected with Rhotekin-RBD beads or PAK-PBD beads, as described in the Materials and methods section. GTP-loaded Rho-GTPase and total Rho-GTPase were detected by Western blotting. (**B**) Band intensities of (**A**) were quantified by densitometry. The ratio of active Rho-GTPase to total Rho-GTPase is shown. The values shown are the means of at least three separate experiments ± SD (error bars); unpaired, two-tailed Student’s t-tests were used to assess significance; *P < 0.05 versus the control, **P < 0.01 versus the control, ^#^P < 0.05 versus the SNAP group, ^##^P < 0.001 versus the SNAP group.

The Rho family of small GTPases, including Rho, Rac, and Cdc42, are small, monomeric G proteins that cycle between an inactive GDP-bound form and an active GTP-bound form. These proteins regulate the actin cytoskeleton, as well as cell migration and proliferation^28^,^29^. Rho regulates actin polymerization, resulting in stress fibre formation and focal adhesion complex assembly. Rac1 and Cdc42 induce the formation of filopodia and lamellipodia, respectively, contributing to the cytoskeletal rearrangements required for cell migration^30^. Rho has been implicated in cell migration, actin organization, focal adhesion formation, and the assembly of adherent junctions and gap junctions in corneal epithelia^31^,^32^. As shown in Fig. 2, the NO donor SNAP not only significantly promoted RhoA activity with ESCs stimulated for 24 h, which was 3.46-fold higher than that of the control (P < 0.001), but also slightly increased Rac1 activity by 1.08-fold compared with the control (P < 0.05). Nevertheless, SNAP did not obviously influence Cdc42 activity. To investigate the pathways by which SNAP regulates Rho GTPases, a cGMP inhibitor (1H-[1,2,4]oxadiazole[4,3-a]quinoxalin-1-one, ODQ) and a PKG inhibitor (Rp-8-pcpt-cGMPs) were used, respectively (P < 0.001, versus the SNAP group). We found that the SNAP-induced activities of RhoA and Rac1 could be blocked by ODQ or by Rp-8-pcpt-cGMPs. Moreover, either the cGMP or the PKG inhibitor could reverse the effect of SNAP on the activation of Rho A and Rac1 (Fig. 2). Meanwhile, similar effects were observed in ESCs stimulated for 10 min (Supplementary Fig. 3); and SNAP exposure significantly activated RhoA and Rac1 in time courses (Supplementary Fig. 2).

### NO stimulates huESC migration via cGMP-Rho GTPase-mediated signal transduction

**Figure 3:**
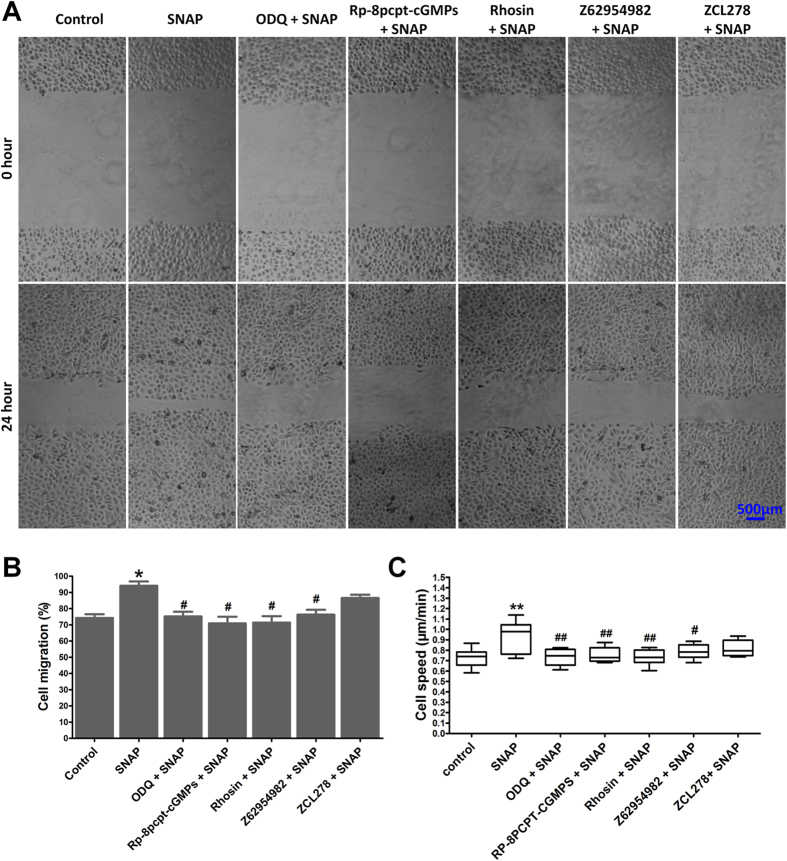
NO regulated huESC migration via cGMP-Rho GTPase signalling. Growth factor-starved ESCs were stimulated with 100 μM SNAP with or without pretreatment for 10 min with 1 μM ODQ (a cGMP inhibitor), 50 μM Rp-8-pcpt-cGMPs (a PKG inhibitor), 30 μM Rhosin (a Rho-specific inhibitor), 50 μM Z62954982 (a Rac1 inhibitor) or 30 μM ZCL278 (a Cdc42 inhibitor). (**A**) Wounds were established in confluent monolayers of huESCs, as described in the Materials and methods section, and the wounded monolayers were cultured for 24 h after treatment. Time-lapse imaging of the wound edges was recorded under an inverted phase microscope. The results are representative of three independent experiments. Scale bars, 500 μm. (**B**) Graphical analysis of (**A**). Cell migration was quantified and graphed as a function of time elapsed vs. percentage of open wound gap. (**C**) Cell motility assay, conducted as described in the Materials and methods section. The values are presented as the mean ± SD of three independent experiments; unpaired, two-tailed Student’s t-tests were used to assess significance; *P < 0.05 versus the control, **P < 0.01 versus the control, ^#^P < 0.05 versus the SNAP group, ^##^P < 0.001 versus the SNAP group.

NO signalling was evaluated during the enhancement of huESC migration. The concentrations of the NO/cGMP/PKG pathway inhibitors were selected based on previous migration experiments^33^. To test directly whether NO facilitates cell migration via its potential downstream target enzymes, 100 μM SNAP was applied in scratch assays (Fig. 3A) and single-cell motility assays (Fig. 3C) after the cells had been treated for ten minutes with a cGMP inhibitor^34^, a PKG inhibitor^35^, a Rho-specific inhibitor (Rhosin), a Rac1 inhibitor (Z62954982) or a Cdc42 inhibitor (ZCL278), respectively. As shown in Fig. 1, SNAP significantly promoted the ESC migration. Furthermore, it was found that these effects could be reduced markedly by ODQ, Rp-8-pcpt-cGMPs, Rhosin or Z62954982 (Fig. 3B,C). However, the Cdc42 inhibitor ZCL278 only slightly blocked SNAP-induced ESC migration (Fig. 3B,C). The same effects were revealed by using specific siRNA-mediated knock-down (Supplementary Fig. 4D,E).

### Effect of NO on the F-actin cytoskeleton in huESCs via cGMP-Rho GTPase signalling

**Figure 4:**
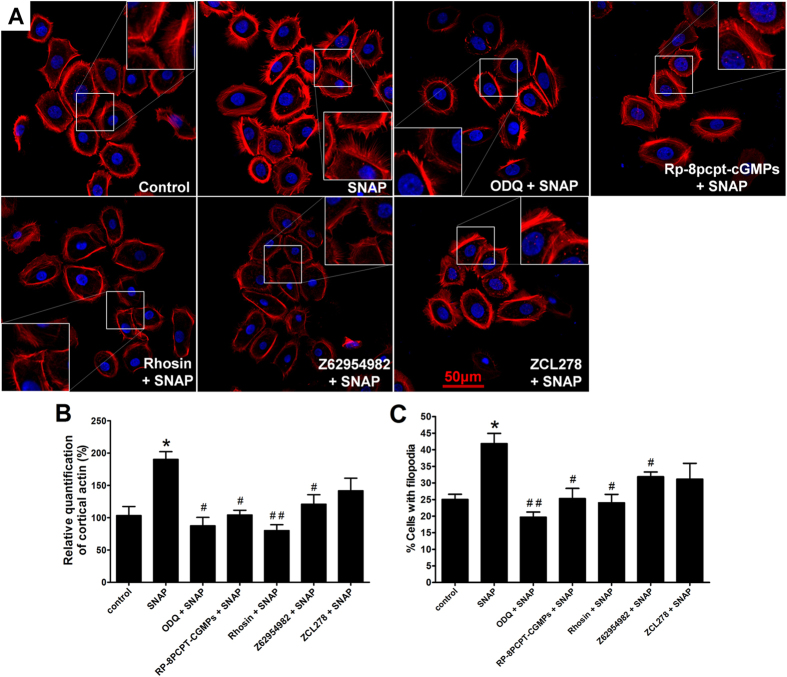
NO regulated huESC F-actin structure via cGMP-Rho GTPase signalling. Growth factor-starved ESCs were stimulated with 100 μM SNAP for 24 h with or without pretreatment for 10 min with 1 μM ODQ (a cGMP inhibitor), 50 μM Rp-8-pcpt-cGMPs (a PKG inhibitor), 30 μM Rhosin (a Rho-specific inhibitor), 50 μM Z62954982 (a Rac1 inhibitor) or 30 μM ZCL278 (a Cdc42 inhibitor). (**A**) F-actin was stained with TRITC-coupled phalloidin, and nuclei were stained with DAPI. Relative quantification of total F-actin: (**B**) cortical actin along cell borders, and (**C**) filopodia actin bundles. The values are presented as the mean ± SD of three independent experiments; unpaired, two-tailed Student’s t-tests were used to assess significance; *P < 0.05 versus the control, **P < 0.01 versus the control, ^#^P < 0.05 versus the SNAP group, ^##^P < 0.001 versus the SNAP group.

Cell migration is a physical process that requires dramatic cytoskeletal rearrangement and changes in cell shape. For efficient movement, these processes must be spatiotemporally coordinated. The morphological changes and physical forces that occur during migration are generated primarily by a dynamic filamentous actin (F-actin) cytoskeleton. F-actin and nuclei were stained using TRITC-coupled phalloidin and DAPI, respectively. An optical trap was integrated with a confocal microscope, thus enabling us to observe F-actin dynamics inside the filopodia and cortical actin along the cell borders (Fig. 4). In the current study, the SNAP treatment resulted in a massive formation of cortical actin bundles and filopodia, which were increased by 1.90-fold and 1.67-fold compared with these in the control group, respectively. In addition, these effects could be reversed by ODQ, Rp-8-pcpt-cGMPs, Rhosin, and Z62954982. However, the Cdc42 inhibitor ZCL278 only slightly decreased the SNAP-induced F-actin dynamics. The same changes were found by using Rho GTPase-specific siRNA (Supplementary Fig. 5).

### The effect of NO on ESC migration via cGMP-Rho GTPase signalling *in vivo*

To detect the effect of NO on ESC migration *in vivo*, a mouse superficial second-degree burn model was established, and the and ESCs were labelled with 5-bromo-2-deoxyuridine (BrdU) *in vivo*. First, A 3-second exposure time to a constant, scalding temperature of 65 °C was used to create the superficial second-degree burn model with adult C57Bl/6 mice, the burn injuries were confirmed using H&E staining and histopathological observation (data not shown).

**Figure 5:**
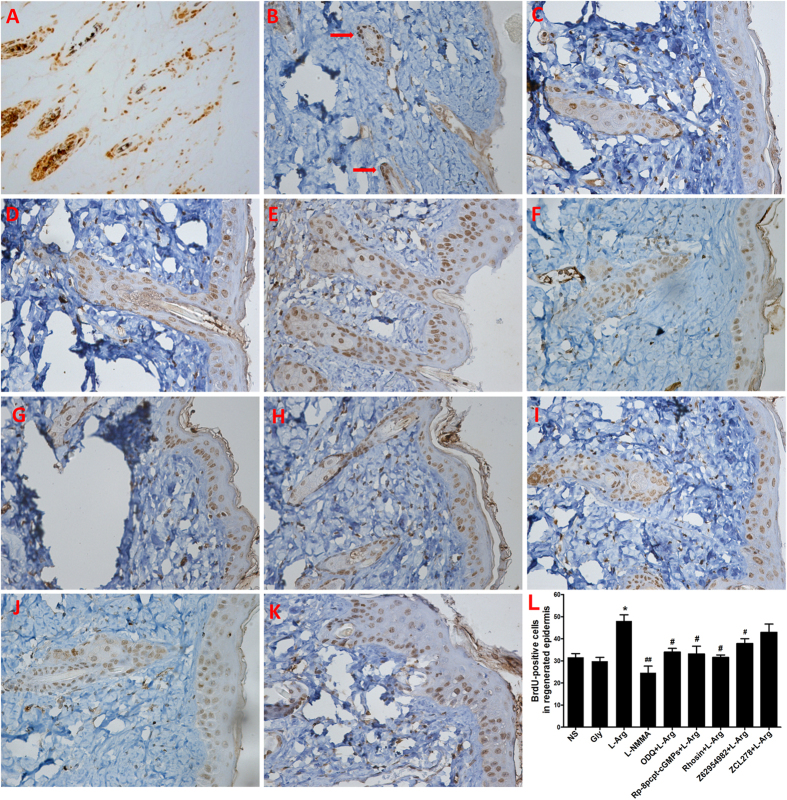
Immunohistochemical analysis of BrdU-labelled cells. (**A**) A paraffin section of the skin of a 3-day-old mouse that had been injected subcutaneously with BrdU twice daily for three days. Note the uniform labelling of all basal epidermal cells and of the entire hair follicle. (**B**) A paraffin section of the skin of a mouse that had been labelled as described in (**A**), followed by a 7-week chase. Note the selective retention of BrdU (tan staining) by the bulge cells; the arrow indicates positive staining. Immunohistochemical staining for BrdU in the mice treated with NS (**C**), Gly (**D**), L-Arg (**E**), L-NMMA (**F**), ODQ + L-Arg (**G**), Rp-8-pcpt-cGMPs + L-Arg (**H**), Rhosin + L-Arg (**I**), Z62954982 + L-Arg (**J**) or ZCL278 + L-Arg (**K**). Slides showing BrdU staining are at 400× magnification. Positive cells appear tan and were present in the regenerated epidermis. At the same magnification, representative images of well-formed, BrdU-positive cells in the regenerated epidermis (**C**–**K**). (**L**) Data analysis was performed using ImagePro Plus software. Columns represent the mean positive cell count in the regenerated epidermis. The data are presented as the mean ± SD of three independent experiments (unpaired, two-tailed Student’s t-tests); *P < 0.05 versus the NS group, ^#^P < 0.05 versus the L-Arg group, ^##^P < 0.001 versus the L-Arg group.

To label ESCs, which present mainly as the label-retaining cells (LRCs) in skin, newborn mice were injected subcutaneously with BrdU twice daily for three days, which generated intense, uniform (nuclear) labelling of virtually all proliferating skin cells, including the bulge cells (Fig. 5A). After a 7-week chase, the only LRCs were detected in the bulge area (Fig. 5B). These findings are consistent with data from previous studies using ^3^H-thymidine (^3^H-TdR), and this indicates that BrdU-labelled cells in the skin are predominantly bulge ESCs^36^,^37^.

L-Arg is a precursor of NO in all mammals^38^. NO is produced when L-arginine (L-Arg) is transformed to L-citrulline by the catalysis of nitric oxide synthase (NOS) in the presence of oxygen and cofactors. The synthesis of NO by NOS isoforms is inhibited by the L-Arg analogue NG-monomethyl-L-arginine (L-NMMA)^39^. In our *in vivo* migration experiments, the numbers of BrdU-positive cells in the regenerated epidermis in the L-Arg group, the L-NMMA group and the saline group were significantly different (Fig. 5C–F), but not Gly group. The number of BrdU-positive cells was enhanced in the presence of the natural NOS substrate L-Arg and suppressed by the NOS inhibitor L-NMMA (Fig. 5L). Moreover, the cGMP, PKG, RhoA and Rac1 inhibitor could abolish the effect of L-Arg-mediated BrdU-positive cells in the regenerated epidermis, but the Cdc42 inhibitor ZCL278 could not (Fig. 5G–K).

## Discussion

The effect of NO on wound healing has been well studied^40^,^41^. However, isolating the disparate effects of NO on wound healing is difficult. Our previous paper showed that NO can enhance the migration of HaCaT keratinocytes *in vitro* via the cGMP/PKG pathway, perhaps by promoting cytoskeleton reorganization^11^. In this study, we found that NO could promote ESC migration and NO accelerates ESC migration via cGMP-Rho GTPase signalling both *in vivo* and *in vitro*.

To detect the effect of NO on ESC migration, we performed migration assays *in vitro*. In the *in vitro* study, the effect of NO on the migration of cultured huESCs in the presence of different concentrations of the NO donor SNAP was detected using a scratch model and time-lapse video microscopy of the motility of living cells. Exogenous NO exerted a biphasic effect on ESC migration, and 100 μM SNAP was the optimal concentration for promoting cell migration. Other authors have also reported that NO exerts a biphasic effect on cell proliferation and migration. Similar to the results of this study, Frank reported^42^ that NO exerts a biphasic effect on HaCaT proliferation, and Kumar^43^ revealed the same phenomenon in HL-60 cells. In our previous study^11^, we found that NO exerts a biphasic effect on HaCaT migration.

The LRC-tracing technique is often used to detect quiescent stem cells *in vivo*. Cell deoxyribonucleic acid (DNA) is labelled by various dyes during DNA synthesis. The underlying premise of this technique is to label cells when all epidermal cells are dividing; labelling is followed by a chase, during which the label is diluted and lost by the more rapidly proliferating cells. This process leaves the slowly cycling cells to be identified via immunohistochemistry. Previous studies have identified LRCs as stem cells by their quiescence in different animal models, with the LRCs in skin being ESCs. Either ^3^H-thymidine (^3^H-TdR) or BrdU can be used as a tracer and be detected by autoradiography or immunohistochemistry, respectively. LRCs in the skin have been reported to be predominantly present in the bulge region of the hair follicle and in the basal membrane, which are the locations of the ESCs^25^,^36^. Furthermore, direct evidence has indicated that the bulge cells generated epidermis in an incision wound model^37^. To establish a model for assaying ESC migration, we labelled LRCs with BrdU instead of ^3^H-TdR because BrdU can be detected more easily. The *in vivo* study showed that the numbers of BrdU-positive cells in the regenerated epidermis were significantly increased by L-Arg and suppressed by L-NMMA (Fig. 5). The positive effects of supplementation with the NO donor arginine, coupled with the negative effects of the NOS inhibitor L-NMMA, provide additional evidence of a key role for NO in ESC migration. The ESC migration process is complex and is accompaniment cell proliferation and differentiation. Therefore, while the method used in this study does not fully elucidate the mechanisms of how NO affects ESC migration, it is clear that ESC migration may be one of the most important issues in wound healing.

Through *in vivo* and *in vitro* experiments, we confirmed that NO may promote ESC migration in wound healing, which indicates that NO may function as a regulator of skin cell motility during wound healing. NO has been recognized as an essential mediator of migration in lung cancer cells and human neural progenitor cells^19^,^44^. Converging experimental evidence from several animal models has suggested that NO participates in the two early developmental processes of cell proliferation and migration^45^,^46^. However, this is the first report to show both *in vitro* and *in vivo* experiments demonstrating that NO can promote ESC migration.

The cGMP synthesizing enzyme sGC is generally accepted as the most important effector for the signalling molecule NO^16^,^17^. The NO/sGC/cGMP/PKG signalling cascade is important in the cardiovascular and nervous systems, as it controls smooth muscle relaxation, synaptic transmission modulation, and cell migration^47^,^48^. However, a brief examination of the signalling pathways implicated in wound healing supports the relevance of the effects exerted by NO in the skin. We found that not only could the NO donor SNAP promote ESC migration and F-actin structure formation but also that this effect could be suppressed by cGMP or PKG inhibitors (Figs 3 and 4). Therefore, the cGMP-mediated signalling pathway may contribute in conjunction with NO to the promotion of ESC migration.

Cytoskeletal reorganization is considered a primary mechanism underlying cell migration^49^. Small G proteins, particularly small GTPases belonging to the Rho family, participate in the processes of cytoskeletal reorganization and cell migration. RhoA, Rac1 and Cdc42 are the primary small GTPases that function in cytoskeletal reorganization^50^. NO can also regulate cytoskeletal architecture, leading to reversible changes in vascular permeability through a Rho GTPase-dependent pathway^51^. In this study, as shown in Fig. 2, SNAP significantly increased RhoA activity 3.46-fold, slightly increased Rac1 activity, and could not obviously affect Cdc42 activity. Treatment with a cGMP or PKG inhibitor blocked the stimulatory effect of NO on the activities of RhoA and Rac1. These results indicate that NO primarily promotes the activation of RhoA and Rac1. Moreover, in the scratch and single-cell motility assays, specific inhibitors of RhoA or Rac1 and RhoA- or Rac1-specific siRNA significantly reduced the cell migration induced by the NO donor SNAP, whereas the effects of the Cdc42 inhibitor and Cdc42-specific siRNA were slight (Supplementary Fig. 4). In addition, increased actin polymerization following SNAP treatment was reversed by treatment with an inhibitor of or siRNA specific to RhoA or Rac; the RhoA inhibitor and RhoA-specific siRNA produced stronger effects. In contrast, the effects of NO on ESC migration via cGMP-Rho GTPase signalling were confirmed by tracing BrdU-labelled cells in a superficial, partial-thickness scald mouse model (Fig. 5). Rho can regulate actin polymerization, resulting in the formation of stress fibres and cortical actin, as well as the assembly of focal adhesion complexes^21^. The Rho-family GTPase Rac can induce the formation of filopodia and lamellipodia, which contribute to the cytoskeletal rearrangements required for cell migration^30^. Sauzeau *et al*. reported that NO was necessary to maintain RhoA expression and RhoA-dependent functions in vascular smooth muscle cells^52^. These data indicate that NO facilitates ESC migration by upregulating the Rho-family GTPases RhoA and Rac1 via the cGMP/PKG pathway.

In conclusion, this study shows that lower concentrations (1–100 μM) of the NO donor SNAP could promote ESC migration, which is likely involved in wound healing. Our study also found that NO could promote ESC migration not only *in vitro* but also *in vivo*, as shown by the ESC migration model involving tracing LRCs marked with BrdU in superficial, partial-thickness scalding. Moreover, the underlying mechanism of NO-induced ESC migration may have been activation of the Rho-family GTPases RhoA and Rac1, but not Cdc42, through cGMP signalling, thereby regulating the reorganization of the F-actin cytoskeleton.

## Materials and Methods

### Isolation and culture of primary huESCs

Primary huESCs were isolated from human foreskins using a modified method of rapid adhesion to collagen IV^53^. These protocols were approved by the Ethics Committee of Southwest Hospital, Chongqing, China. All experiments were performed in accordance with ethical guidelines and regulations. The foreskin tissue was obtained from the redundant prepuce of patients 12–20 years old who provided informed consent. In brief, after being disinfected and rinsed three times with PBS, the foreskin tissues were cut into approximately 0.5-cm^2^ pieces and digested with 0.25% Dispase II (Roche, Basel, Switzerland) overnight at 4 °C. Then, the epidermis was separated carefully and digested with 0.25% trypsin for 10 min at 37 °C. The cells were collected by filtration centrifugation and washing with PBS. Finally, the isolated cells were suspended in keratinocyte serum-free medium (K-SFM, Invitrogen, California, USA) supplemented with epidermal growth factor (0.1–0.2 ng/ml), bovine pituitary extract (20–30 mg/ml), CaCl_2_ (0.05 mM) and 100 IU/ml of streptomycin and penicillin. Then, 5 ml of isolated suspended cells were seeded at a concentration of 2 × 10^5^/ml in 25-mm dishes that had been coated overnight with collagen IV (100 μg/ml, Sigma, Saint Louis, USA). The cells were incubated for 10 min at 37 °C and 100% humidity in an atmosphere of 5% CO_2_ in air. Non-adherent cells were then immediately rinsed off. The adherent cells were further cultured with fresh medium at 37 °C and 100% humidity in an atmosphere of 5% CO_2_ in air. The medium was changed every other day. When the cells of passages 1 and 2 grew to 60–70% confluence, they were digested with 0.25% trypsin +0.02% EDTA (Sigma, Saint Louis, USA) for 5–10 min. The cells at the second passage were used for identification as well as for other experiments.

### Double immunofluorescence staining for β1 integrin and CK19

Cells from the second passage were seeded and cultured on coverslips precoated with type IV collagen for 24 h. The cultured cells were washed with PBS and fixed with 70% methanol in acetone. After being washed three times with PBS, the coverslips were blocked for 1 h with 1% BSA (Sigma, Saint Louis, USA) at room temperature (RT). Then, the cells were incubated sequentially with mouse anti-human β1 integrin (1:100, Santa Cruz, California, USA) at 4 °C overnight and with TRITC-conjugated donkey anti-mouse secondary antibody (1:500, Invitrogen, California, USA) for 1 h at RT. Next, the cells were stained with rabbit anti-human CK19 (1:100, Sigma, Saint Louis, USA) for 12 h at 4 °C and FITC-conjugated goat anti-rabbit secondary antibody (1:500, Invitrogen) for 1 h at RT. Finally, the stained cells were examined using a laser-scanning confocal fluorescence microscope (Leica, Munich, Germany).

### Colony-forming efficiency (CFE) assay

Suspended cells from the second passage were aliquoted into six-well plates precoated with collagen IV at a density of 1000 cells in 2 ml of K-SFM medium per well and were cultured at 37 °C with 100% humidity and 5% CO_2_. The medium was changed every 2 days for 2 weeks. Then, the cells were fixed in 4% paraformaldehyde (PFA) for 30 min and stained with 0.1% crystal violet stain solution (Sigma) for 15 min at RT. The cells were imaged using a digital camera, and the number of colonies was counted under an inverted microscope (Olympus, Japan). Each colony consisted of at least 32 cells. The CFE was defined as the ratio of the number of colonies to the initial number of cells inoculated.

### Flow cytometry analysis

Flow cytometry analysis was performed using a BD FACSAria Flow Cytometer (BD Biosciences, San Jose, CA). Separated cells were dual-stained with a PE-conjugated CD71 monoclonal antibody (No. sc-7327 PE) and a FITC-conjugated a6 integrin monoclonal antibody (No. sc-19622 FITC) from Santa Cruz Biotechnology (Santa Cruz, California, USA). Normal mouse IgG1-PE (No. sc-2866) and normal rat IgG2a-FITC (No. sc-2831) from Santa Cruz, which were used as isotype-matched negative controls, were used at equal concentrations and subjected to the same labelling protocol. Labelling reactions were performed in the dark for 1 h at 4 °C. The fluorescence intensity was determined by flow cytometry from a minimum of 1 × 10^4^ cells. Experiments were repeated three times using the same conditions and settings.

### Cell viability after treatment with the NO donor SNAP

Cell viability was measured using a Cell Counting Kit-8 (CCK-8) (Dojindo, Kumamoto, Japan). The ESCs were cultured in triplicate (100 μl/well) in 96-well plates, treated with different concentrations of SNAP for 24 h, and further incubated with 10 μl of CCK-8 for 4 h at 37 °C. CCK-8-positive cells were considered viable, and viability was expressed as a percentage compared with the control cells.

### Transient transfections

The specificities of the siRNA pools were confirmed with the following independent siRNAs: RhoA, 5′-CACAGUGUUUGAGAACUAUTT-3′ and 5′-GCUAGACGUGGGAAGAAAATT-3′; Rac1, 5′-GGAACUAAACUUGAUCUUATT-3′ and 5′-CCUUUGUACGCUUUGCUCATT-3′; Cdc42, 5′-UGAGAUAACUCACCACUGUTT-3′ and 5′-AGAUCUAGUUUAGAAAACATT-3′ (Invitrogen). siGLO RNA-induced silencing complex-free control siRNA was purchased from Thermo Fisher Scientific. In total, 50 nM siRNA was transfected into huESCs using Lipofectamine 2000 (Invitrogen) according to the manufacturer’s protocol. The cells were then incubated in K-SFM for 24 h before further experimentation.

### Western blotting

The stimulated cells were lysed with lysis buffer and resolved on a 10% SDS-PAGE gel before being transferred onto nitrocellulose membranes. The membranes were blocked at RT with Tris-buffered saline containing 3% BSA. Then, the membranes were incubated at RT with various anti-primary antibodies and probed with rabbit anti-mouse horseradish peroxidase (HRP)–conjugated secondary antibody. Bands were visualized using enhanced chemiluminescence.

### Cell migration assay

ESC migration was evaluated using an *in vitro* wound-healing model, as described in the literature^54^, with some modifications. In brief, second-passage huESCs were cultured nearly to confluence in six-well plates precoated with type IV collagen. Mitomycin C (Sigma, Saint Louis, USA) was added to a final concentration of 4 μg/ml and maintained for 2 h to stop cell proliferation^55^. Then, a cell-free area in each well was created by scraping the cell monolayer with a sterile 10-μl plastic pipette tip. The medium and non-adherent cells were aspirated off, and the plates were rinsed twice with PBS. Then, various concentrations of SNAP (Sigma, Saint Louis, USA) in fresh culture medium were added to the wells, with three wells used per condition. Time-lapse imaging of the wounds was conducted using an inverted phase microscope over a 24-h period. The residual areas between the migrating ESCs were quantified using ImageJ software (NIH, National Institutes of Health, USA). The migration rates were calculated using the following equation: (initial area - residual area)/initial area × 100%.

### Cell motility assay

All single-cell motility assays were performed in 24-well plates (Corning Costar, Cambridge, MA) as previously described^56^. In brief, second-passage ESCs were seeded at a density of 2 × 10^4^ cells/1 ml/well in 24-well plates precoated with type IV collagen and allowed to grow for 48 h. The cells were maintained at 37 °C in a humidified atmosphere containing 5% CO_2_. The cell motilities were recorded for 24 h as phase-contrast time-lapse movies using a Zeiss Axiovert 135T inverted microscope. Images were obtained at 5-min intervals using AQM Advance 6 Kinetic Acquisition Manager (Medical Solutions, PLC) and were subsequently analysed with Adobe ImageReady. Cell speed was determined by tracing the cell periphery every hour for 24 h with ImageJ tracking software (NIH, National Institutes of Health, USA).

### Pull-down assay

The activities of RhoA, Rac1 and Cdc42, which belong to the Rho family of small GTPases, were measured using pull-down assay kits (Cat. BK034/BK035/BK036, Cytoskeleton, Denver, USA) according to the manufacturer’s instructions. In brief, PAK-PBD beads with a fusion protein consisting of the p21-binding domain of Pak1, which specifically binds to the GTP-bound Rac1 and/or Cdc42 proteins^57^, but not to the inactive forms of Cdc42-GDP and/or Rac1-GDP, was used for the precipitation of Cdc42-GTP and Rac1-GTP. GTP-γS and GDP were used as positive and negative controls, respectively. Phosphorylated Pak1, which is a substrate for Cdc42 and Rac1, was used as an indicator of Cdc42 and Rac1 activity. Pak1 binding to PAK-PBD beads induces autophosphorylation and activation. Rhotekin-RBD beads were used as an indicator for active RhoA. The beads were washed three times with lysis buffer, and the bound GTP-Rho was detected by immunoblot analysis with mouse anti-human monoclonal Cdc42 antibody (1:250), mouse anti-human monoclonal Rac1 antibody (1:500), or mouse anti-human monoclonal RhoA antibody (1:500), followed by goat anti-mouse HRP-conjugated secondary antibody (1:5000). An enhanced chemiluminescence detection method was applied to detect the RhoA/Cdc42/Rac1 signals.

### F-actin cytoskeleton assay

Actin cytoskeleton reorganization was assessed via phalloidin staining, as previously reported^11^. In brief, second-passage ESCs were seeded and cultured on coverslips precoated with type IV collagen for 24 h. The cells were starved of growth factors for 12 h. Then, the cells were fixed in 4% PFA and permeabilized with 0.1% Triton X-100/1x PBS for 3 min at RT. Blocking was performed with 10% BSA/PBS (1 h, RT), and the cells were subsequently incubated with TRITC-coupled phalloidin (1:200, Sigma, Saint Louis, USA) for 20 minutes and DAPI (5 μg/mL, Sigma) for 5 minutes. Images of fluorescently labelled specimens were acquired using an LSM 510 laser-scanning confocal microscope (Carl Zeiss, Inc.) equipped with LSM 510 software and immersion lenses (Carl Zeiss, Inc.). Cortical actin and filopodia in 100 cells on each slide were randomly analysed under a Zeiss fluorescence microscope. LSM 510 and ImageJ software were employed to determine the percentage of cells with cortical actin and filopodia, according to previously described methods^58^,^59^. Experiments were repeated on three independent occasions.

### Animals

C57Bl/6 mice were purchased from The Experimental Animal Department of Third Military Medical University, China. All animals in this study were used in accordance with ethical standards, and the animal protocols were approved by the Institutional Animal Care and Use Committee of Third Military Medical University. The animals were individually raised in plastic cages under standard conditions (temperature, 25 °C; relative humidity, 50%; and circadian rhythm, 12 h). The animals were given standard autoclaved rodent chow and water ad libitum and were acclimated to the facility for 1 week before the experiment.

### ESC migration assay in a mouse model of burn injury

Based on Taylor’s method, the slow-cycling cells in mouse skin were labelled with BrdU^37^. In brief, neonatal C57Bl/6 mice were injected intraperitoneally (i.p.) with BrdU (50 mg/g body weight, Sigma) beginning on day 3 after birth, twice daily (8 a.m. and 5 p.m.) for 3 days. Cells retaining the label after 7 weeks were identified as LRCs and confirmed with BrdU immunohistochemical staining. The positive cells, or LRCs, in the skin were considered ESCs^37^. At this time, each mouse was subjected to a superficial partial-thickness burn injury on the dorsum.

The superficial partial-thickness burn model was performed as described in the literature, with some modifications^60^. In brief, seven-week-old mice were anesthetized with an i.p. injection of 0.1% sodium pentobarbital at 10 μl per gram of body weight, followed by interscapular hair removal. According to the results of a preliminary experiment, a metal plate (Shandong Academy of Medical Science, Jinan) with a diameter of 1.5 cm and weight of 0.5 kg was used to induce superficial partial-thickness burns on mouse dorsal skin. The metal plate was heated to 65 °C and placed evenly on the shaved mouse dorsum between the scapulae for 3 sec. The wound was confirmed histopathologically as a superficial, second-degree, partial-thickness burn.

The injured mice were divided randomly into the following nine groups: L-Arg; Gly; L-NMMA; normal saline (0.9% sodium chloride, NS); ODQ + L-Arg; Rp-8-pcpt-cGMPs + L-Arg; Rhosin + L-Arg; Z62954982 + L-Arg; and ZCL278 + L-Arg. Immediately after being injured, the mice in each group were injected (0.05 ml/g body weight, i.p.) with L-Arg (4 mg/ml in NS, Sigma), Gly (4 mg/ml in NS, Sigma), L-NMMA (0.4 mg/ml in NS, Beyotime China), normal saline (as control), or (0.05 ml/g body weight, i.p.) L-Arg (4 mg/ml in NS) together with the following five inhibitors, respectively: ODQ (a cGMP inhibitor, 0.4 mg/ml in NS), Rp-8-pcpt-cGMPs (a PKG inhibitor, 0.4 mg/ml in NS), Rhosin (a Rho-specific inhibitor, 0.4 mg/ml in NS), Z62954982 (a Rac1 inhibitor, 0.4 mg/ml in NS) or ZCL278 (a Cdc42 inhibitor, 0.4 mg/ml in NS). Each group contained five mice. Subsequently, the mice were placed on a heating pad to maintain body temperature and were then housed in individual cages, as described for the superficial partial-thickness burn model. After 48 h, the wounds were biopsied, fixed in paraformaldehyde and sectioned at a thickness of 5 μm. The BrdU-positive cells were detected using immunohistochemistry. Five random fields in each section were imaged using a microscope at 400× amplification. The total and BrdU-positive cells in the re-epithelialization area in each field were counted using Image-Pro Plus software (Media Cybernetics).

### Statistical analyses

The data are expressed as the mean ± SD of the indicated number of observations. Comparisons between two groups were performed using unpaired, two-tailed Student’s t-tests. One-way analysis of variance (ANOVA) was applied for single-cell speed assay using different concentrations of SNAP. A general univariate linear model was used to analyze the migration assay results after treatment with SNAP for different times. P values < 0.05 were considered statistically significant.

## Additional Information

**How to cite this article**: Zhan, R. *et al*. Nitric oxide promotes epidermal stem cell migration via cGMP-Rho GTPase signalling. *Sci. Rep.* **6**, 30687; doi: 10.1038/srep30687 (2016).

## Supplementary Material

Supplementary Information
