# Supplementary material for: Nitric oxide promotes epidermal stem cell migration via cGMP-Rho GTPase signalling
Source: Sci Rep. 2016 Jul 29;6:30687. doi: 10.1038/srep30687 (PMC4965828; doi:10.1038/srep30687)
Supplement: Supplementary Information [file srep30687-s1.pdf]

Nitric Oxide promotes epidermal stem cells migration via cGMP-Rho GTPase signaling

Supplementary Figures

Rixing Zhan, Weifeng He, Fan Wang, Zhihui Yao, Jianglin Tan, Rui Xu, Junyi Zhou, Yuzhen Wang, Haisheng Li, Jun Wu, Gaoxing LUO

Supplementary Figure Legends and Figures

Supplementary Fig. 1. **Cell survival induced by the NO donor SNAP at different concentrations.** Cell death was quantitated using a Cell Counting Kit-8 according to the manufacturer's instructions. Percentage cell survival was calculated by dividing the optical density of treated cells by the optical density of untreated control cells, and multiplying by 100. The survival of the control cells was set at 100%; * P<0.05 versus control, **P<0.01 versus the control. Data shown are the means and standard deviations of duplicate experiments on samples from three different donors (unpaired, two-tailed Student's t-tests).

Supplementary Fig. 2. **Time course of NO-induced Rho GTPase activation**. ESCs were incubated with 100 μM SNAP for the indicated periods of time. (A) The cell lysates were subjected to pull-down and Western blot analysis using the RhoA antibody. (B, C) Band intensities of (A) were quantified by densitometry, and the ratios of active RhoA to total RhoA (B) and active Rac1 to total Rac1 (C) are shown. The data shown are from a typical experiment involving three representative experiments. Unpaired, two-tailed Student's t-tests were used to assess significance; *P 0.05 versus the control, **P<0.01 versus the control.

Supplementary Fig. 3. **NO activated Rho GTPase via cGMP/PKG in 10 min.** Growth factor-starved ESCs were stimulated with 100 μM SNAP for 10 min with or without pretreatment for 10 min with 1 μM ODQ, as a cGMP inhibitor, or 50 μM Rp-8-pcpt-cGMPs, as a PKG inhibitor. (A) Active Rho-GTPase was detected with Rhotekin-RBD beads or PAK-PBD beads, as described in the Materials and methods section. GTP-loaded Rho-GTPase and total Rho-GTPase were detected by Western blotting. (B) Band intensities of (A) were quantified by densitometry. The ratio of active Rho-GTPase to total Rho-GTPase is shown. The values shown are the means of at least three separate experiments. The values are presented as the mean ± SD of three independent experiments. Unpaired, two-tailed Student's t-tests were used to assess significance; *P<0.05 versus the control, **P<0.01 versus the control, $^{\#}$P<0.05 versus the SNAP group, $^{\#\#}$P<0.001 versus the SNAP group.

Supplementary Fig. 4. **NO regulated huESC migration via cGMP-Rho GTPase signalling tested by specific siRNA-mediated knock-down.** (A) A representative Western blot demonstrating two distinct siRNAs per gene of RhoA, Rac1 and Cdc42. (B) Quantification of Western blots represented in A; **P<0.001 versus the siGLO control. (C) After transfection with specific siRNA for 50 nM, a scratch assay was performed using confluent monolayers, as described in the Materials and methods sections, and the wounded monolayers were cultured for 24 h after being treated with 100 μM SNAP. (D) Graphical analysis of (C). Cell migration was quantified and graphed as a function of time elapsed vs. percentage of open wound gap. (E) The cell motility assay was conducted as described in the Materials and methods section. After being transfected with specific siRNA, huESCs were treated with 100 μM SNAP for 24 h. The data are expressed as the mean ± SD of values from three independent experiments, each performed in duplicate (n=3). Unpaired, two-tailed Student's t-tests were used to assess significance; **P<0.001 versus the control, $^{\#}$ <0.05 versus the siGLO + SNAP group, $^{\#\#}$P<0.001 versus the siGLO + SNAP group.

Supplementary Fig. 5. NO regulated huESC F-actin structure formation via cGMP-Rho GTPase signalling, as indicated by specific siRNA-mediated knock-down. (A) After being transfected with specific siRNA, huESCs were treated with 100 μM SNAP for 24 h. F-actin was stained with TRITC-coupled phalloidin, and nuclei were stained with DAPI, as described in the Materials and methods section. Quantification of total F-actin: (B) cortical actin along cell borders, and (C) filopodia actin bundles. The values are presented as the mean ± SD of three independent experiments. Unpaired, two-tailed Student's t-tests were used to assess significance; $^{*}P<0.05$ versus the control, $^{\#}P<0.05$ versus the siGLO + SNAP group， $^{\#\#}P<0.001$ versus the siGLO + SNAP group.

Supplementary Figures

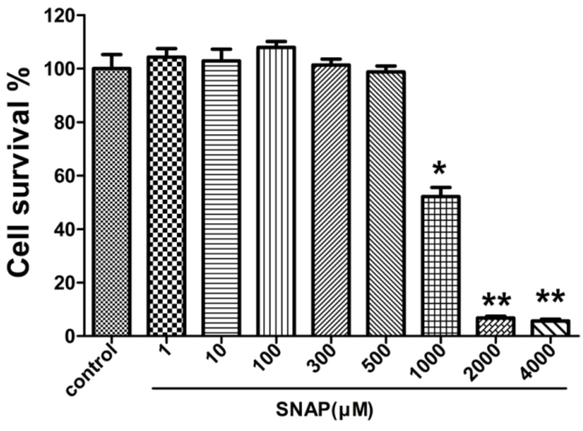

Supplementary Figure 1

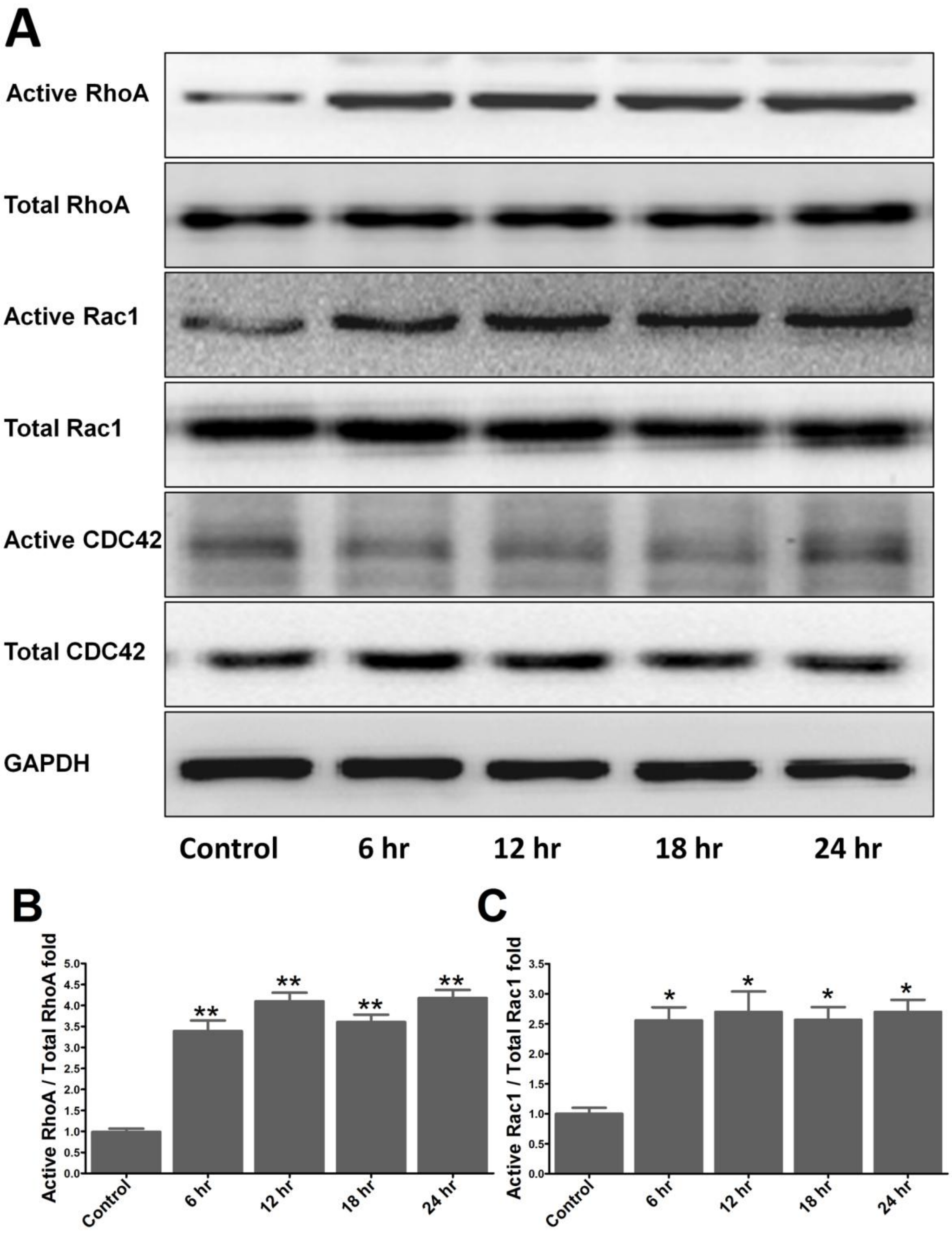

Supplementary Figure 2

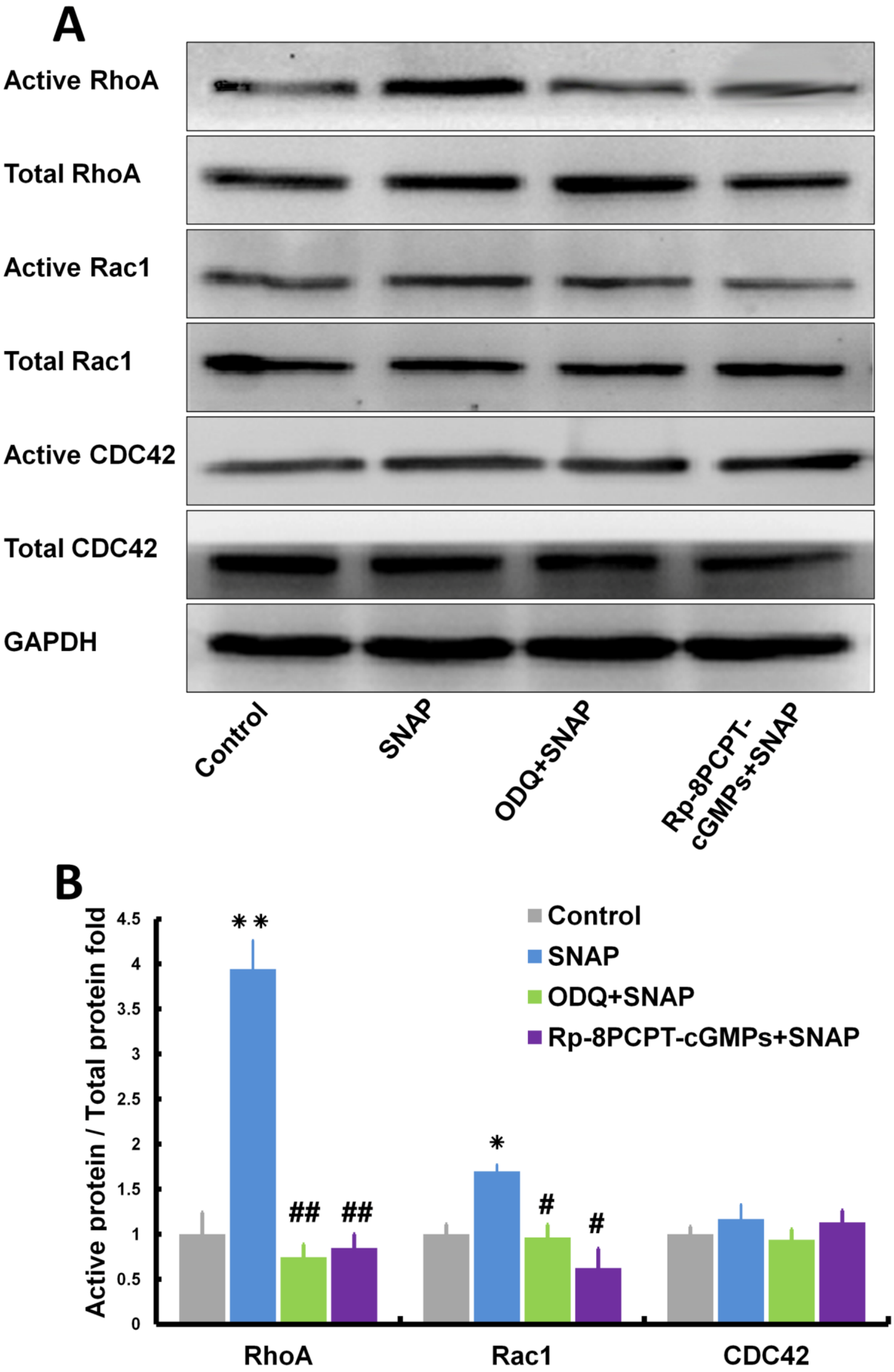

Supplementary Figure 3

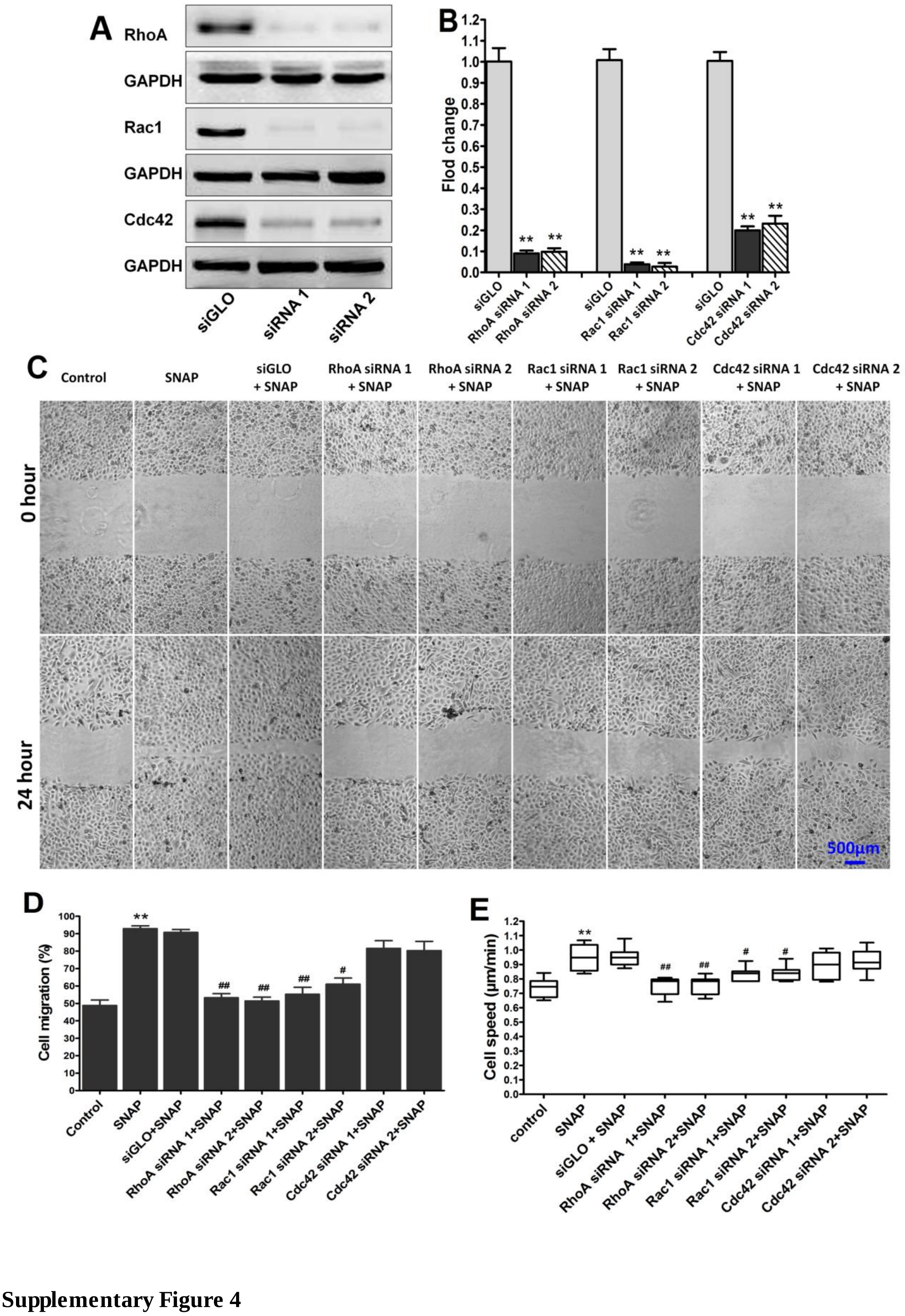

Supplementary Figure 4

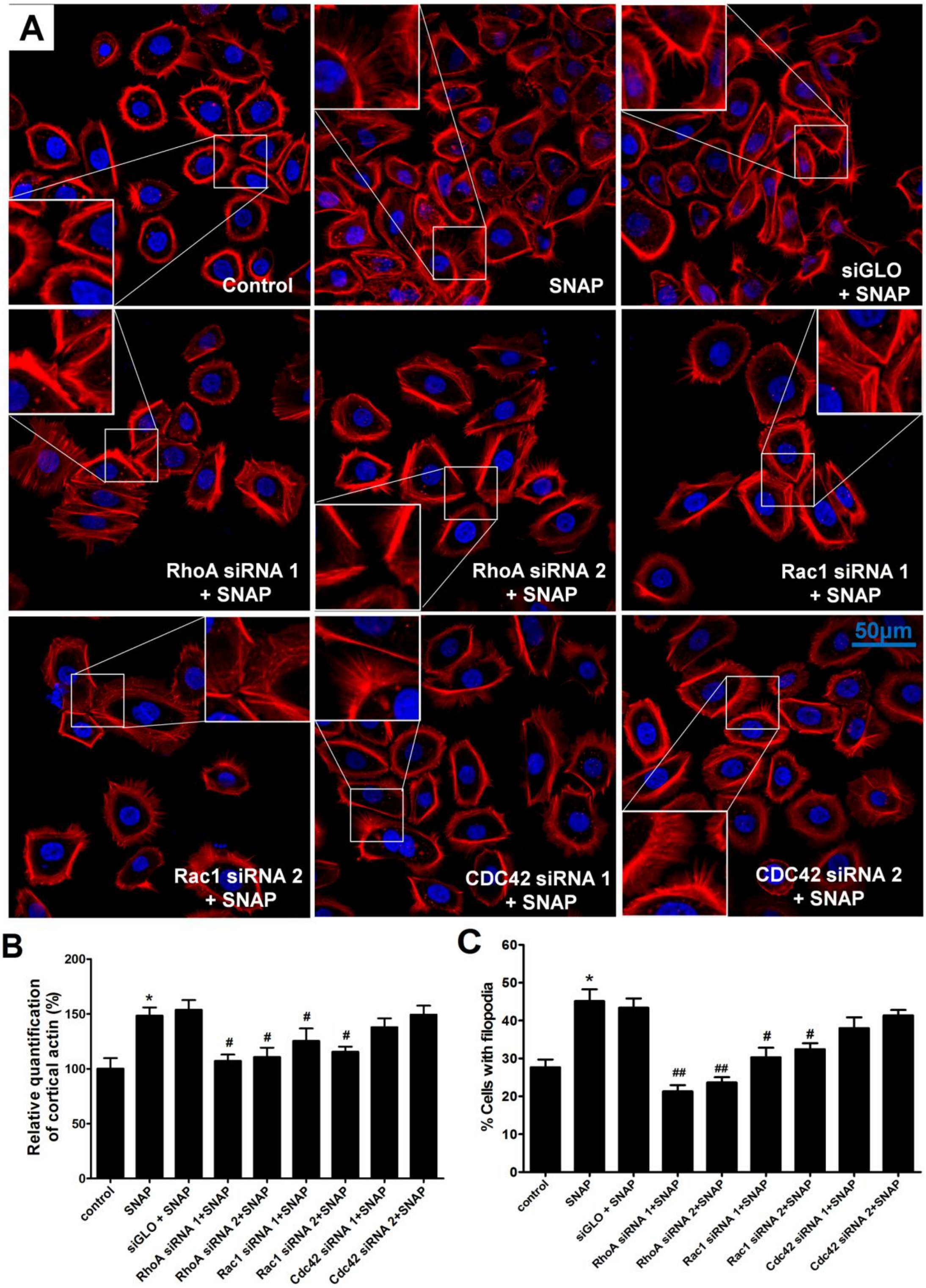

Supplementary Figure 5
